# ICP35 Is a TREX-Like Protein Identified in White Spot Syndrome Virus

**DOI:** 10.1371/journal.pone.0158301

**Published:** 2016-06-27

**Authors:** Panapat Phairoh, Thana Suthibatpong, Triwit Rattanarojpong, Nujarin Jongruja, Saengchan Senapin, Kiattawee Choowongkomon, Pongsak Khunrae

**Affiliations:** 1 Department of Microbiology, Faculty of Science, King Mongkut’s University of Technology Thonburi, Bangmod, Bangkok, 10140, Thailand; 2 Department of Physics, Faculty of Science, King Mongkut’s University of Technology Thonburi, Bangmod, Bangkok, 10140, Thailand; 3 National Center for Genetic Engineering and Biotechnology, National Science and Technology Development Agency, Pathumthani, 12120, Thailand; 4 Center of Excellence for Shrimp Molecular Biology and Biotechnology, Mahidol University, 272 Rama VI Road, Bangkok, 10400, Thailand; 5 Department of Biochemistry Faculty of Science Kasetsart University, Bangkok, 10900, Thailand; Uppsala University, SWEDEN

## Abstract

ICP35 is a non-structural protein from White spot syndrome virus believed to be important in viral replication. Since ICP35 was found to localize in the host nucleus, it has been speculated that the function of ICP35 might be involved in the interaction of DNA. In this study, we overexpressed, purified and characterized ICP35. The thioredoxin-fused ICP35 (thio-ICP35) was strongly expressed in *E*. *coli* and be able to form itself into dimers. Investigation of the interaction between ICP35 and DNA revealed that ICP35 can perform DNase activity. Structural model of ICP35 was successfully built on TREX1, suggesting that ICP35 might adopt the folding similar to that of TREX1 protein. Several residues important for dimerization in TREX1 are also conserved in ICP35. Residue Asn126 and Asp132, which are seen to be in close proximity to metal ions in the ICP35 model, were shown through site-directed mutagenesis to be critical for DNase activity.

## Introduction

White spot syndrome virus (WSSV) is a causative agent of white spot disease in shrimp. The virus can have an enormous impact on global Penaied shrimp farming as the infection could result in 100% cumulative mortality in farmed shrimps within 7 days [1–3]. WSSV is a rod-shaped closed circular double stranded DNA virus comprising of 305,107 bp and classified as the species type *Whispovirus* in a new virus family *Nimaviridae*. Nucleotide sequence analysis reveals that the dsDNA genome of WSSV encodes 181 open reading frames (ORFs) which are predicted to be able to translate into viral proteins [4–6].

ICP35, previously known as VP35, was first identified in 2001 as Western blot analysis initially indicated the presence of the protein in nucleocapsid fraction [7]. In 2013, it was however confirmed by the same group that the protein was actually a nonstructural protein. Henceforth, the protein was renamed as ICP35 [8]. Dual luciferase assay has shown that ICP35 was transcribed by internal ribosome entry site (IRES) dependent mediation and highly expressed in shrimp cells during viral infection [8]. The *ICP35* transcript could be detected in WSSV infected shrimp, *Penaeus monodon*, at 2 h p.i. (hours post infection) and the expression level significantly increased at 18 h p.i, indicating its potential role in the infection process. *ICP35* encodes a 687 bp open reading frame coding the protein with a molecular mass of around 35 kDa [7, 8]. Amino sequence analysis suggested that ICP35 had two nuclear localizing signals (^24^KRKR^27^ and ^53^KRPR^56^). Observation under fluorescent microscope also revealed that ICP35 was localized in the nucleus of sf9 cells. It has been speculated that ICP35 may serve protein-DNA interaction important in viral replication [7], but there has been no direct evidence to support whether or not the ICP35 protein can interact with DNA.

In this study, recombinant ICP35 was expressed and purified from *E*. *coli* expression system. Functional characterization of ICP35 revealed that the protein contains nuclease activity. Structural prediction suggested that ICP35 is a nuclease whose structure is adopted from TREX1. Site-directed mutagenesis was also performed to identify the amino residues critical for DNase activity in ICP35.

## Materials and Methods

### Bacterial Strains, Plasmids and Shrimp

*Escherichia coli* BL21 (DE3) strains from Novagen were used for protein expression. The DNA cloning was performed using pET15bThio, a pET15b (+) from Novagen which was modified to have thioredoxin and TEV cleavage site encoding sequence inserted upstream of the multiple cloning region. A linearized pET15bThio vector was used in a DNase activity analysis experiment. *E*. *coli* were cultured in 2XYT broth (SIGMA).

### Cloning of ICP35

Secondary structure prediction of ICP35 suggested that the first 31 residues at N-terminal of ICP35 resided in a highly flexible region which could impose a difficulty in the protein expression in *E coli*. In this study, those 31 residues were removed, therefore the ICP35 boundary used in this study is starting from amino residue 32 to 228. The ICP35 encoding sequence was amplified using WSSV genome as the template for PCR amplification. Specific primers for amplification of the gene encoding ICP35 were ICP35-F (**CGC** GGA TCC GTA AAG GTG AAA GTG GAA GTA), ICP35-R (**ACA CCG** CTC GAG TTA CCA ACA AGG ATC ATC) for forward and reverse primer respectively. All of the primers contained flanking non-complementary sequences (bold type), including appropriate restriction sites (underlined) so that the desired restriction sites would be included in amplicons. The PCR reaction (50 μl) consisted of 100 ng of WSSV template, 0.2 μM of each primer, 0.2 μM of dNTP mix, and 5 U of Taq DNA polymerase (Stratagene) in 1× PCR reaction buffer. The reaction mixture was performed under the following conditions: denaturation at 94°C for 5 minutes; 25 cycles of 95°C for 30 s, 55°C for 30 s and 72°C for 1 minute, with a final extension at 72°C for 10 minutes. To construct ICP35 expression plasmids, the *Bam*HI-*XhoI* amplicon containing an ICP35 encoding gene was cloned in-frame into pET15bThio. The DNA sequencing analysis confirmed the in-frame insertions in the recombinant vectors.

### Expression and Purification of Recombinant ICP35 Protein

The ICP35 expression vector was transformed into *E*. *coli* BL21. After transformation, the bacteria were cultured in 1 L of 2XYT broth containing 50 μg/ml of ampicillin and incubated at 37°C with shaking at 200 RPM until OD_600_ reached 0.8. The expression of the thio-ICP35 was induced by adding IPTG into the culture broth to the final concentration of 0.5 mM and left to shake slowly overnight at 15°C and 100 RPM. Bacterial cells were harvested by centrifugation and resuspended in 35 ml of lysis buffer (20 mM Tris-base, 0.3 M NaCl, 15 mM imidazole, 0.5% (v/v) Triton X-100). The cell suspension was sonicated 4 times with the amplitude of 40% for 8 seconds and centrifuged at 20,000x g, 4°C for 20 minutes to collect supernatant. The supernatant was then subjected to Ni-NTA column (Qiagen). The column was washed 5 times with lysis buffer to clean off the unbound proteins. The His-tagged ICP35 that bound in the column was eluted with 5 ml of elution buffer (20 mM Tris-base, 0.15 M NaCl, 0.2 M imidazole) for 5 times. To remove the thioredoxin tag, thio-ICP35 was transferred to TEV cleavage buffer (20 mM Tris-base, 50 mM NaCl, pH 8.0) by using PD-10 Desalting Columns (GE-Healthcare Life Science). 1.5 mg of TEV protease was added in the 5 mg protein solution and incubate at 4°C for 16 hours. The thioredoxin tag released from TEV cleavage was eliminated by flowing the protein mixture through Ni-NTA column and collecting the flow-through solution. The TEV-cleaved ICP35 was then concentrated down to 1 ml by using Amicon Ultracentrifuge filter (Merck Millipore) with the molecular weight Cut-off at 10,000 Da and then purified by Size-exclusion chromatography on HiLoad^TM^ 16/600 Superdex^TM^ 200 column (GE-Healthcare Life Science) using the DNA binding buffer (20 mM Tris-HCl, pH 8.0, 200 mM NaCl and 5 mM MgSO_4_) as a running buffer. For Western blotting analysis, the protein was separated in SDS-PAGE (12%, w/v) followed by transferring to polyvinylidene difluoride (PVDF) membrane (GE Healthcare Life Science). The membrane was subsequently blocked with 5% skimmed milk for 2 hours. Penta·His HRP Conjugate antibody (Qiagen) was used at concentration of 1:5000 with 2.5% skim milk. Detection was performed with Clarity Western ECL Substrate (Biorad) and visualized in ImageQuant LAS 500 chemiluminescence detection (GE Healthcare Life Science). Immuno detection of TEV-cleved ICP35 were performed by incubation of the blot in polyclonal rabbit anti-ICP35 serum diluted 1:500 in 2.5% skim milk. Subsequently, Goat anti-Rabbit IgG HRP conjugated (Thermo Fisher Scientific) was used at concentration 1:2000 and detection was carry out in the same method as above.

### DNase Activity Analysis

DNase activity analysis for investigation of protein-DNA interaction were conducted using a protocol modified from Wang HC and Yue Li [9, 10]. The linearized pET15bThio vector was prepared by single digestion with *Bam*HI restriction enzyme (New England BioLabs) and heat inactivated at 60°C for 30 minutes followed by purification by Miniprep (Qiagen). The reactions were performed in the DNA binding buffer containing 300 ng of linearized pET15bThio vector and thio-ICP35 proteins at different concentrations, ranging from 0–50 pmol. The protein-DNA mixtures were incubated at 25°C for 30 minutes before loading onto 1.0% agarose gels. Electrophoresis was performed using TBE buffer followed by ethidium bromide staining and observed under UV. Control proteins used in this experiment was thioredoxin produced in *E*. *coli* harboring pET15bThio vector by the protocol mentioned above. Inhibition of DNase activity was carried out in the DNA binding buffer containing EDTA at concentration of 10 mM.

### Homology Modeling of ICP35 Protein

To characterize the function of ICP35, automated homology modeling was performed by using the SWISS-MODEL server [11]. Crystal structure of three prime repair exonuclease I (TREX1) (PDB ID: 3b6o with 2.10 Å resolution) was used as a template, All pictures of the ICP35 model were created in PyMOL. To validate the model, the internal molecular mechanics (MM) energy and implicit solvent solvation energy have been calculated using AMBER99 force field parameters and generalized-Born surface energy approximation (GB/SA) method for the structures of ICP35 monomer and ICP35 dimer after performing an energy minimization in explicit solvent. The pairwise GB/SA energy decomposition was performed by using a python script freely available in the AmberTools15 package to elucidate the energetic contribution of the conserved salt bridge and hydrogen bonding residues.

### Mutagenesis of ICP35

Single point mutations were introduced by PCR-base mutagenesis, using the QuikChange Site-Directed Mutagenesis procedure (Stratagen) by which a target amino acid residue was replaced with Alanine (A). The forward mutagenic primers were N126A (ATC AAA CTG AAA GCC CCC CTA AGG GAA), D132A (CTA AGG GAA CAT GCG ATG GCA GTT TCA), D181A (AAA GAG TTT GGC GCC ATG GAG ATT GGA TCT) and reverse primers were reverse complement with the forward strand. The thio-ICP35 construct was used as the template for mutant version. PCRs for single amino acid mutations were run for 20 cycles of 30 s at 95°C and 1 minutes at 50°C, followed by 4 minutes at 70°C. The sequences of the plasmids were confirmed by DNA sequencing. The expression and purification of the mutants were carried out as above.

## Results

### The Expression of ICP35

**Fig 1:**
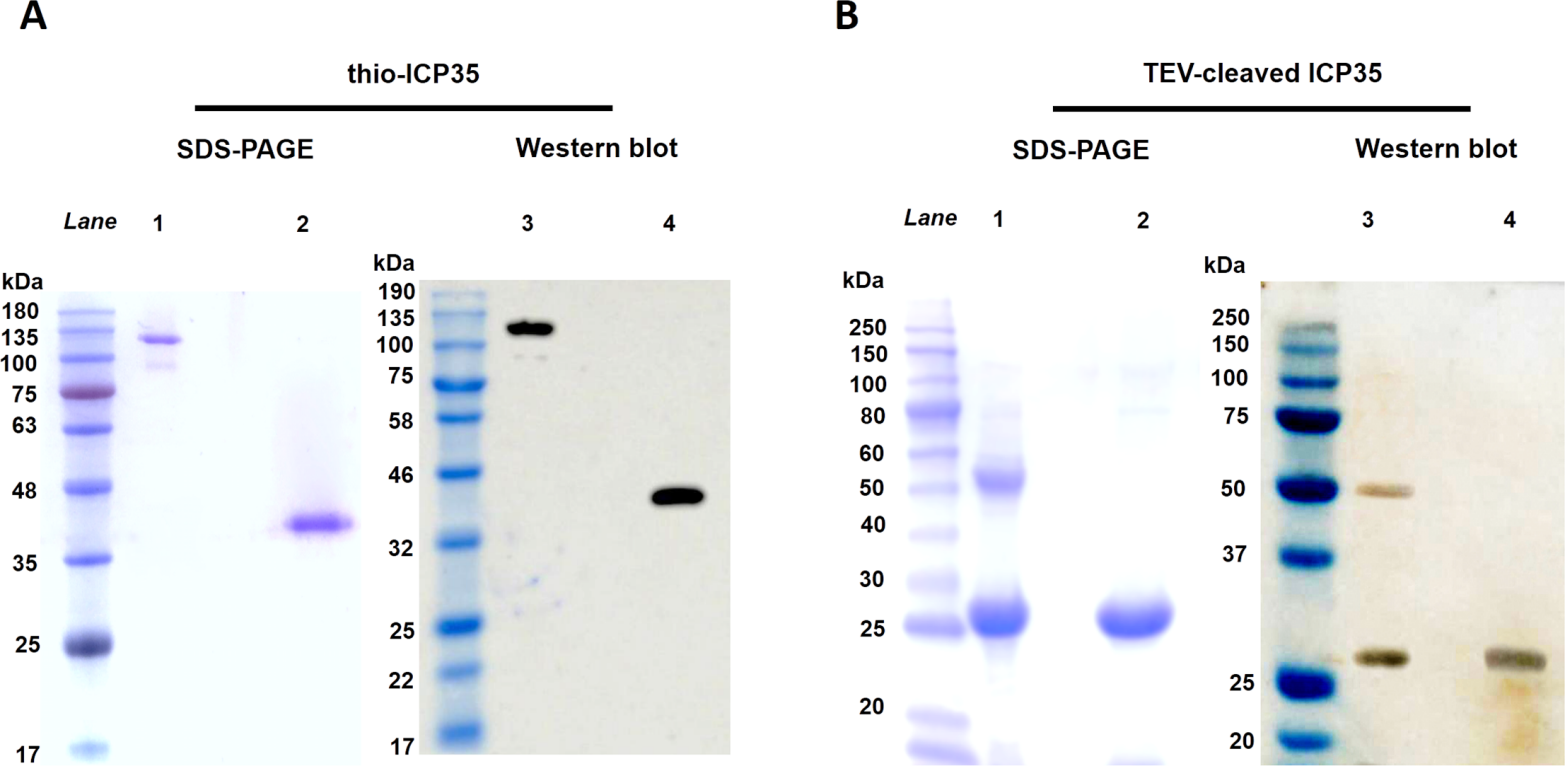
Coomassie-stained SDS-PAGE gels and Western blot analysis of ICP35 proteins. **(A)** thio-ICP35 was observed on SDS-PAGE and Western blot with (lane 2 and 4) and without (lane 1 and 3) being boiled prior to loading. **(B)** TEV-cleaved ICP35 was observed on SDS-PAGE and Western blot with (lane 2 and 4) and without (lane 1 and 3) being boiled prior to loading. Oligomerization of ICP35 could be observed in both before and after the removal of thioredoxin fusion tag.

Secondary structure prediction of ICP35 suggested that the first 31 residues at N-terminal of the full-length ICP35 were highly flexible which could cause the protein to be unstable in solution. We therefore decided to remove those 31 residues and tested expression in *E*. *coli* BL21. As shown in Fig 1A, the expression of thio-ICP35 was observed. Interestingly, the thio-ICP35 appeared on SDS-PAGE at the molecular weight of around 130 kDa when it was loaded without boiling (Fig 1A, lane 1). Once the protein was boiled, it appeared on gel at the molecular weight of around 38 kDa (Fig 1A, lane 2). Western blot analysis confirmed that the protein was the expression product as it could react with the anti-His antibody (Fig 1A, lane 3–4). We suspected that ICP35 might be able to form itself into a larger complex through oligomerization and that the oligomerization was not due to the effect of thioredoxin, we then removed thioredoxin from thio-ICP35 by TEV cleavage and concentrated the TEV-cleaved ICP35 to the concentration of 10 mg/ml to induce the oligomerization. In Fig 1B, TEV-cleaved ICP35 was also seen to produce a protein band corresponding to the molecular weight of 50 kDa when loaded unboiling (Fig 1B, lane 1). It could also be seen that the 50 kDa band was completely broken down to 25 kDa when boiled (Fig 1B, lane 2). As the mass difference between the boiled and unboiled TEV-cleaved ICP35 is double, this suggested that TEV-cleaved ICP35 might be able to form dimer. However, when the different mass between the heavy (130 kDa) and the light (38 kDa) band of thio-ICP35 was considered, it was found that the mass of the heavy band was approximately triple the mass of the light band. Nevertheless, several proteins expressed in fusion with thioredoxin appear on SDS-PAGE bigger than their expected mass [12–14]. This might also be the case for thio-ICP35.

Since the ICP35 dimer could be easily destroyed by boiling, it could be said that the dimerization of ICP35 may not require disulfide linkage to stabilize the dimer. Western blot analysis by using rabbit anti-ICP35 confirmed both the dimer and monomer band were ICP35 protein (Fig 1B, lane 3 and 4). It is worth noting that all of the thio-ICP35 could as a complex (Fig 1A, lane 1). However, once thioredoxin had been removed, only a part of TEV-cleaved ICP35 was seen to form complex (Fig 1B, lane 1). This could be explained by the fact that thioredoxin may help in stabilizing protein conformation [15, 16] given that the functional form of the protein is required for dimerization. Removal of thioredoxin might yield an instable protein whose structural conformation is less favorable for functioning and this might also be the reason why we had to concentrate the TEV-cleaved ICP35 up to 10 mg/ml to induce dimer formation.

### DNase Activity Analysis of ICP35

Amino acid sequence analysis of ICP35 revealed that the protein possesses two NLS motifs. In addition the protein was also shown to localize in the nucleus of the SF9 cells [7]. Since then, it has been speculated that ICP35 may have a function involved in interacting with DNA. In this experiment, we investigated the interaction between ICP35 and DNA by incubating them together and observed on agarose gel.

**Fig 2:**
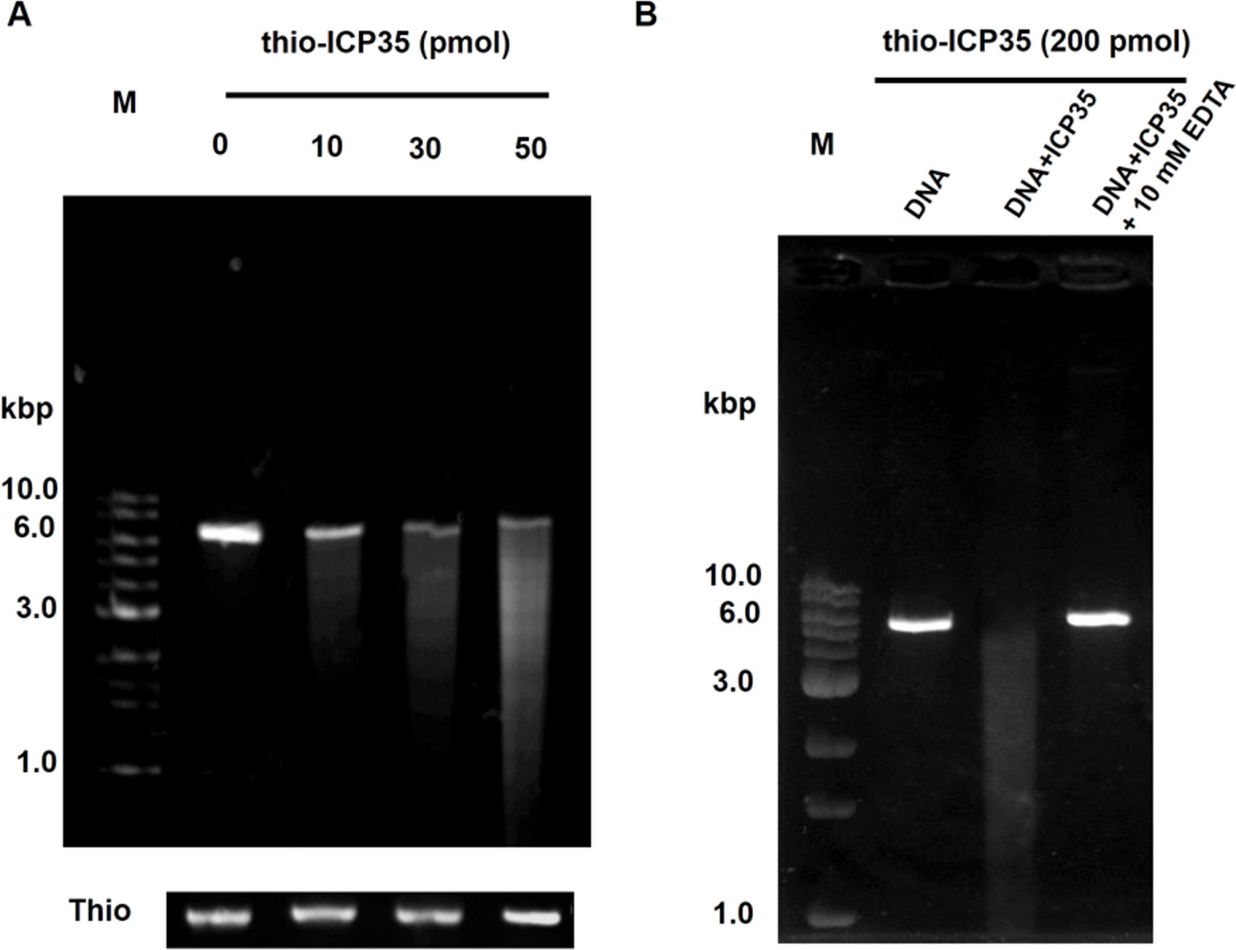
DNase activity by agarose gel electrophoresis for investigating the interaction between ICP35 and DNA. **(A)** The digestion of DNA by thio-ICP35 was observed in dose-dependent. The DNA digestion was more pronounced over the concentration of ICP35. **(B)** The digestion of DNA by thio-ICP35 was inhibited by 10 mM EDTA.

As shown in Fig 2A, 300 ng of linearized pET15bThio vector was incubated for two hours with thio-ICP35. We kept the thio-ICP35 at a low concentration ranging from 0 to 50 pmol in order to observe the effect of thio-ICP35 concentration without saturating the reaction. It was clearly seen that the DNA was degraded and appeared as a smear down along the lane (Fig 2A). We then hypothesized that thio-ICP35 protein might contain DNA digestion activity that causes the degradation of the DNA. In addition, we observed that the DNA digestion appeared more pronounced with an increase in concentration of thio-ICP35, strongly indicating that the DNA digestion was due to the activity of thio-ICP35. Having seen that thio-ICP35 can digest DNA seems to suggest that ICP35 might be a nuclease. EDTA, a universal nuclease inhibitor, was then used to test if it could inhibit thio-ICP35 from digesting DNA. Thio-ICP35 was incubated with DNA in the DNA binding buffer containing 10 mM EDTA and the result was shown in Fig 2B. It was seen in Fig 2B that EDTA could inhibit the activity of thio-ICP35 as the DNA stayed intacted even after incubated with thio-ICP35 at the concentration of 200 pmol for two hours. Through our results, ICP35 was shown for the first time that it might function as a nuclease.

**Fig 3:**
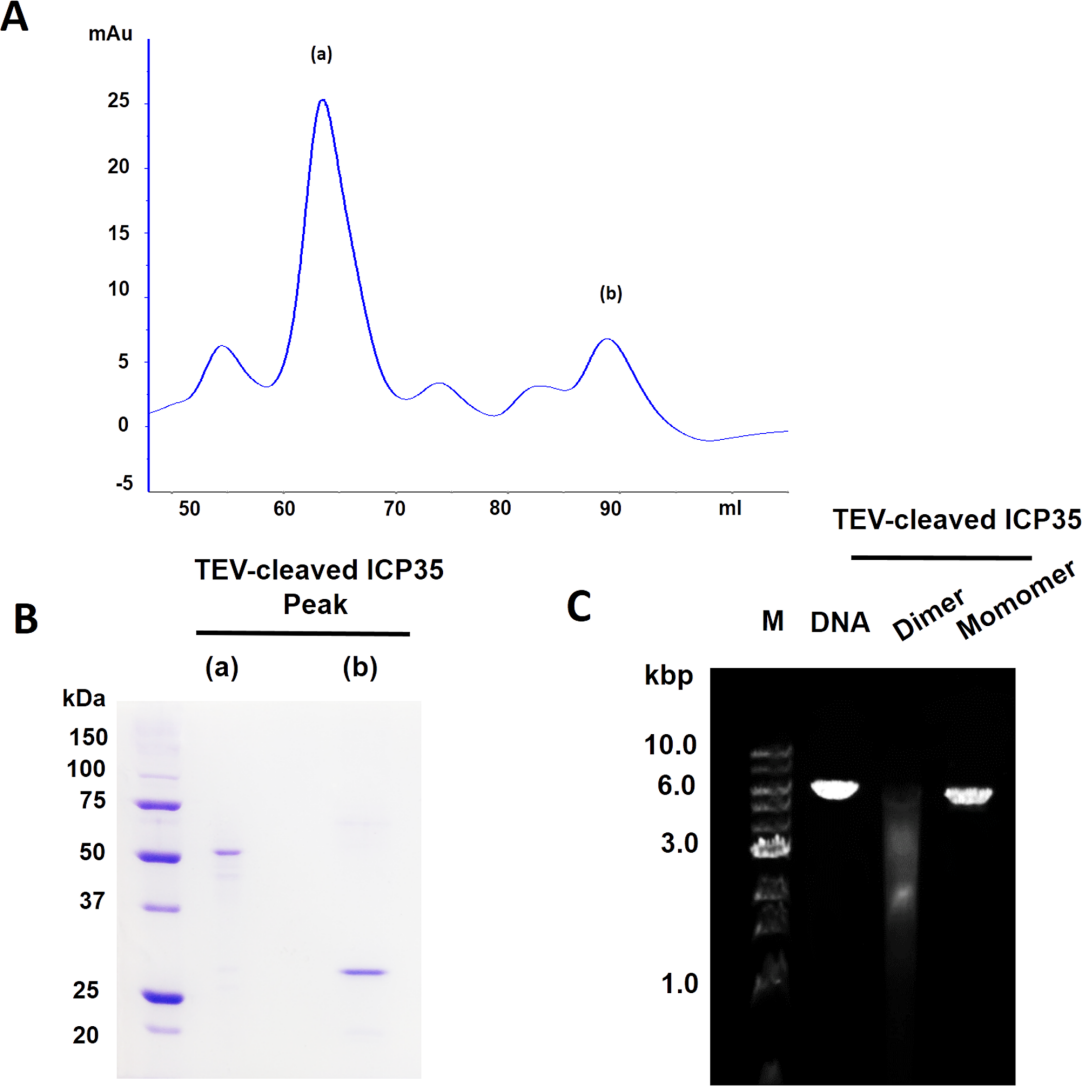
Separation of dimer and monomer form of TEV-cleaved ICP35 by size-exclusion chromatography. **(A)** Size-exclusion chromatogram of TEV-cleaved ICP35 showed two major peaks, (a) and (b) corresponding to the fraction 66 and 90 ml respectively. **(B)** SDS-PAGE analysis revealed that the faction peak (a) and (b) was consisted of dimer and monomer form of TEV-cleaved ICP35 respectively. **(C)** DNase activity analysis showed only the dimer form of TEV-cleaved ICP35 digests DNA.

Since the thio-ICP35 was used in the DNase activity analysis, this has raised the question if the DNase activity observed in thio-ICP35 could be the artifact caused by conformational modification by thioredoxin. To prove that the DNase activity observed in thio-ICP35 was not due to the effect from thioredoxin fusion, we used thioredoxin as a control and it was confirmed that the DNA digestion observed in the experiments was not due to thioredoxin (Fig 2A). We also investigated the DNase activity in TEV-cleaved ICP35 both in monomer and dimer form (Fig 3A and 3B). We found that TEV-cleaved ICP35 could also be able to digest DNA and interestingly only the dimer form of TEV-cleaved ICP35 could perform DNA digestion (Fig 3C). This strongly indicates that ICP35 possesses an intrinsic DNase activity and that the DNase activity observed in thio-ICP35 was not an artifact from thioredoxin fusion. Since thio-ICP35 was more stable in solution than TEV-cleaved ICP35, thio-ICP35 was then used in subsequent experiments.

### Structural Model of ICP35

**Fig 4:**
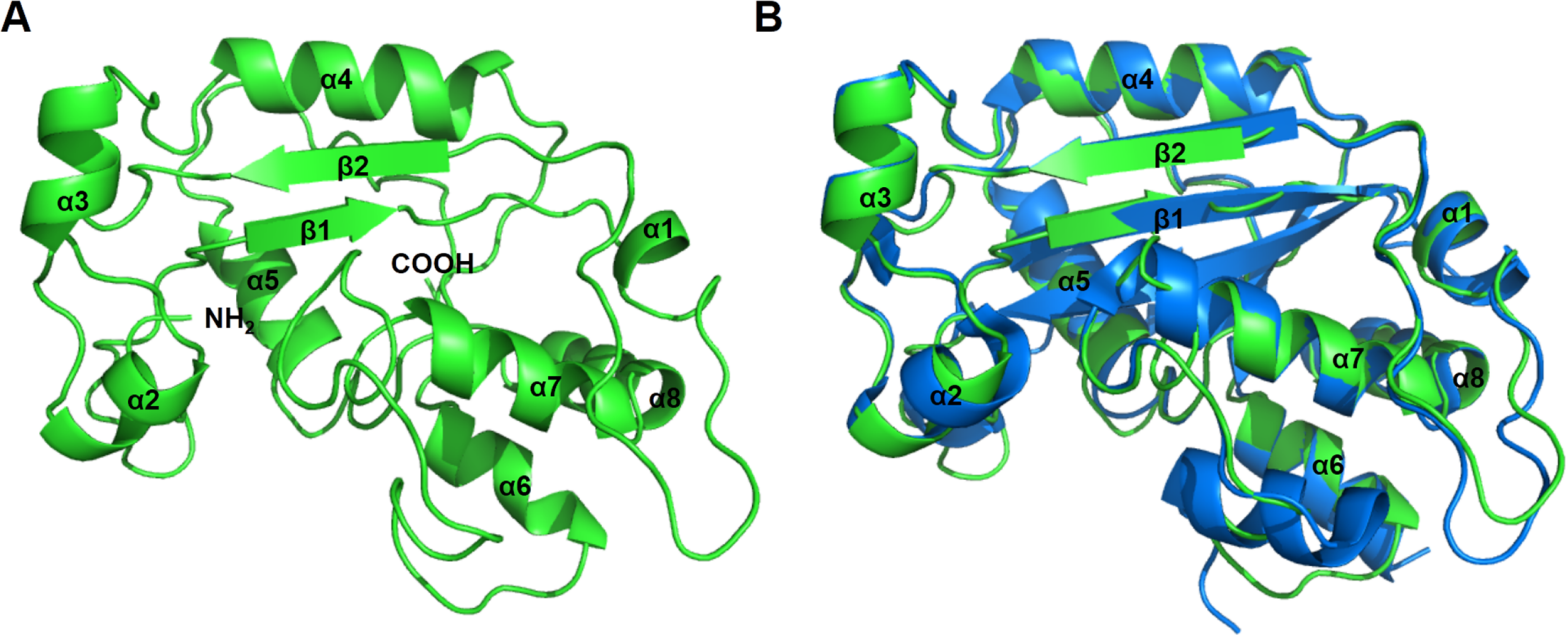
(A) Structural model of ICP35 built from residue 18 to 213 using three prime repair exonucleaseI (TREX1) from *Mus musculus* (PDB code: 3b6o) as a template. (B) The superimposition of ICP35 model (green) with the TREX1structure (blue) gave r.m.s.d. at 0.821 Å.

In order to gain insights into the function of ICP35, the structural model of ICP35 was built by using Swiss model server [11]. Initially, the server failed to search for a proper template as it could produce only incomplete models that cover a few regions of ICP35. We then manually selected the template using important properties of ICP35 as selective criteria. In this respect, the template must be a nuclease whose size is comparable to the size of ICP35 and able to form itself into dimer. We found that the three prime repair exonuclease I (TREX1) satisfied those criteria and hence was used as a template for modeling of ICP35. Among several TREX proteins used in modeling, TREX1 from *Mus musculus* (PDB code: 3b6o) was found to be the most suitable template as it yielded the ICP35 model with the highest in Q-mean sore (8.57), and model coverage (85.52%) that reflects the model is reliable when used for describing the structure of ICP35. As TREX1 is a member of DnaQ-like 3'-5' exonuclease family, we also tried using other structures, TREX2 (PDB: 1y97), ribonuclease T (PDB: 2f96) and exosome complex exonuclease RRP6 (PDB: 2hbk), from DnaQ-like 3'-5' exonuclease family to build ICP35 models. We found that none of the templates produce a better result than that obtained from TREX1 as they failed to produce a model with coverage larger than 50%. This could imply that the structure of ICP35 is more closely related to the TREX1. The structural model of ICP35 is shown in Fig 4A. The obtained model covers the region of ICP35 from residue 18 to 213. It was seen in the model that ICP35 contains 8 alpha helixes and 2 anti-parallel beta sheets. Superimposition of ICP35 model onto the structure of TREX1 shows that the secondary structure elements of ICP35 are well aligned into the structure of TREX1 with r.m.s.d. of 0.821 Å. (Fig 4B). This suggests that ICP35 might adopt protein folding similar to the TREX1.

**Fig 5:**
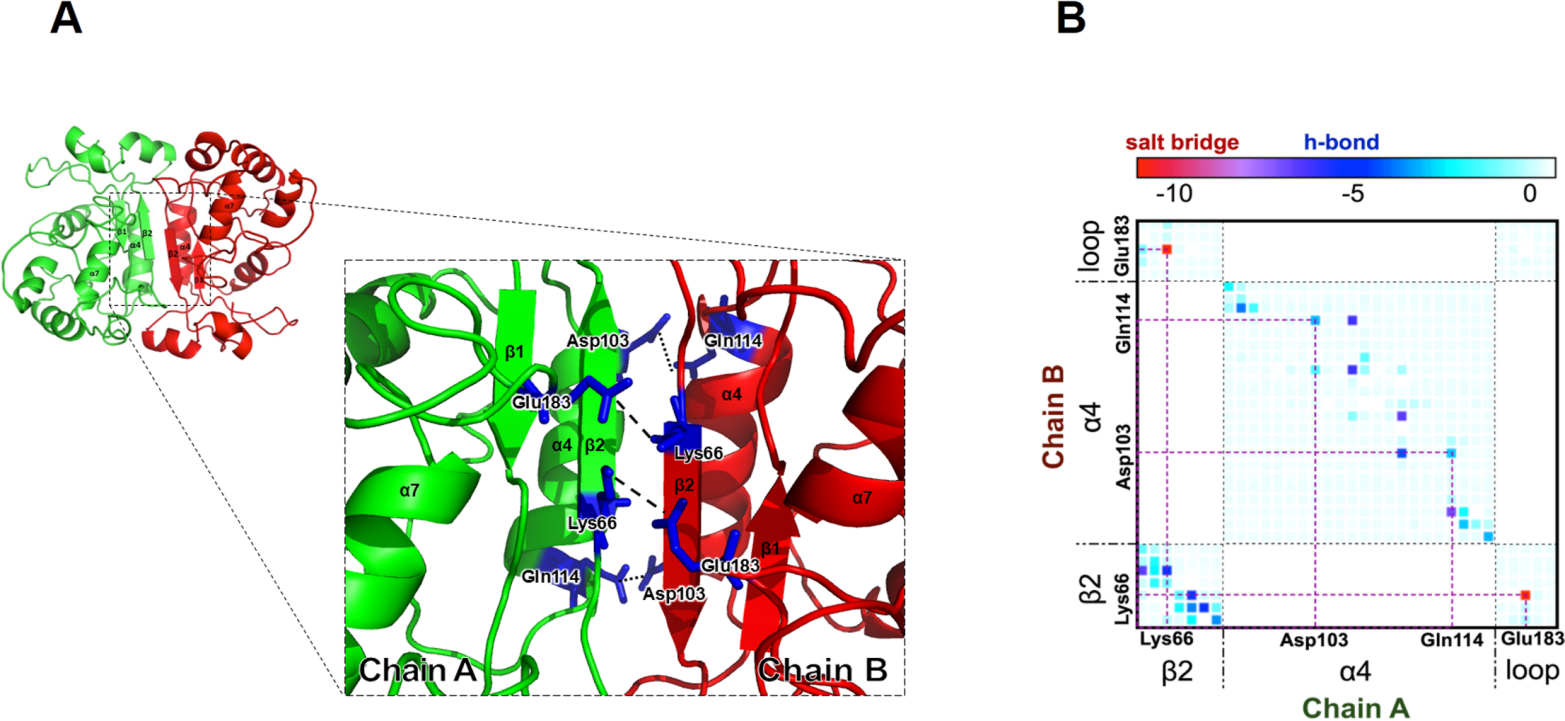
(A) Dimeric structure of ICP35 (monomers shown in green and red). The residues colored in blue, Lys66, Asp103, Gln114 and Glu183 were believed to be critical for ICP35 dimerization by forming intermolecular salt bridges (dash lines) and hydrogen bonds (dot lines). (B) 2D map of decomposed MM-GB/SA energy calculations for each pair of residues from Chain A and Chain B. Purple dashed lines indicate the conserved residues, also found in TREX1 proteins.

TREX1 is known to be able to form a homodimer to create an active form for dsDNA degradation [17, 18]. We therefore used the structural information from TREX1 dimer to model the structure of the ICP35 dimer as shown in Fig 5A. Our homology modeling suggests that ICP35 could form a dimer through the same mechanism as seen in TREX1 that is each ICP35 protomer forms a dimer by positioning its active site on opposite edges. The propensity of dimer formation was verified by an MM-GB/SA energy calculated from the ICP35 structures obtained by homology model, in which the total MM-GB/SA energy of the dimer was less than that of two individuals ICP35 protomers (see Table 1). This suggests that ICP35 tend to form dimer to stably exist in solution. Several conserved residues playing an important role in dimerization in TREX1 such as Lys66, Asp103, Gln114 and Glu183 are also found to reside in the equivalent positions at the interface of the ICP35 dimer. In the model of ICP35 dimer, the protomers are seen interacting with each other along the beta strand (β2), alpha helix (α4) by the anti-parallel direction. The conserved residue at the interface, such as, Asp103 and Gln114 are located on the helix α4, while Lys66 is located on the strand β2 and Glu183 is located on a loop, having their side chains facing toward the interface. Local contributions to binding energy were then calculated from the pairwise decomposition of MM-GB/SA energy. Fig 5B shows the map of MM GB/SA energy between each pair of residues from chain A and chain B at the dimerization interface, in which we consider the strand β2 (residues 64–70), the helix α4 (residues 96–117) and the loop (residues 181–185) containing the conserved E183 residue. Pair interaction between the conserved Lys66 and Glu183 exhibits the largest energy contribution and can be classified as a ‘salt-bridge’. Another pair of conserved residues, Asp103 and Gln114, forms a hydrogen bond, which stabilizes the α4 helix.

**Table 1:** Generalized-Born surface energy approximation (GB/SA) calculated from ICP-35 structures in protomer and dimer forms.

Energy type	2 × ICP-35 protomer (kcal/mol)	ICP-35 dimer (kcal/mol)	ΔG (kcal/mol)
MM	2 × (4944)	17815	7927
GB/SA	2 × (-11654)	-31869	-8651
Total	-13420	-14054	-634

**Fig 6:**
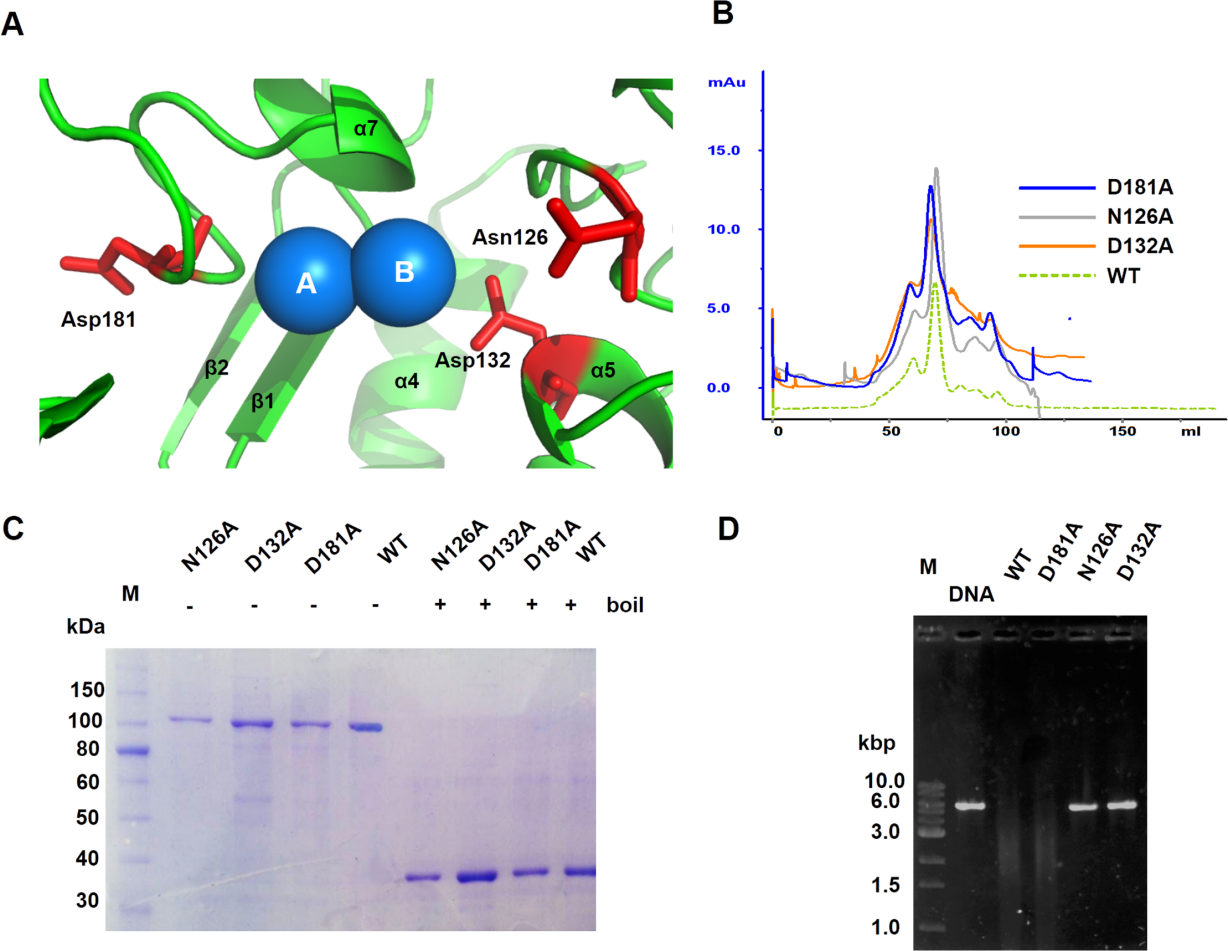
(A) Asn126 and Asp132 are believed to be a metal binding residue for ICP35 as they are positioned closely to the metal ions. (B) Size-exclusion chromatogram of thio-ICP35 wild type and mutants. (C) SDS-PAGE analysis of thio-ICP35 wild type and mutants. (D) DNase activity analysis for investigating the interaction of thio-ICP35 both wild type and the mutants with DNA.

It was shown inTREX1 that residue Asp18, Glu20, Asp130 and Asp200 are critical for nuclease activity as the mutation on these residues results in a drastic decrease in nuclease activity [19, 20]. These residues correspond to the DEDD motif which is a unique element responsible for metal ion binding exclusively found in DnaQ-like 3'-5' exonuclease family to which TEX1 belongs. However, this motif is lacking in ICP35. When we superimposed ICP35 structure onto the structure of TREX1 in complex with DNA (PDB: 3b6o) and analyzed the region that is believed important for metal binding, we found that the metal binding pocket of ICP35 is similar in size with TREX1, hence two metal ions were placed into the pocket of ICP35. In this pocket, residue Asn126, Asp132 and Asp181 of ICP35 were found in close proximity to metal ions as seen in Blue sphere (Fig 6A). In addition, Asn126 and Asp132 are also conserved in the equivalent position of the TREX1. We then performed site-directed mutagenesis to generate N126A, D132A and D181A mutants of ICP35 and tested for their role in nuclease activity.

As shown In Fig 6, N126A, D132A and D181A mutants were successfully expressed and purified as they were shown to have the same size-exclusion chromatogram and SDS-PAGE profile as seen in the wild type thio-ICP35 (Fig 6B and 6C). All the mutants together with the wild type thio-ICP35 were tested for their ability to digest DNA in DNase activity analysis as described previously. It was found that N126A and D132A were not able to digest the DNA while D181A was still able to perform DNA digestion the same as the wild type (Fig 6D). Our mutagenesis results confirm the role of residue Asn126 and Asp132 in nuclease activity in ICP35. In TREX1, DEDD motif was shown to be positioned around the metal ions and was critical for nuclease [19]. Considering the position of Asn126 and Asp132 in ICP35 model, that they sit closely to the metal ions, together with the results from mutagenesis, it could be said that ICP35 uses Asn126 and Asp132 to serve as a metal binding residue instead of using the DEDD motif. Since the DEDD motif is missing in ICP35, our results seem to suggest that ICP35 may adopt the structure from TREX1 but the mechanism underlying nuclease activity of ICP35 may be different. For residue Asp181, it was seen resided in a long flexible loop and facing out from metal ions in ICP35, it might be possible that the loop is moving away from the metal ions so that Asp181 does not play any role in nuclease activity.

## Discussion

The function of ICP35 from WSSV has been a mystery for more than a decade since its discovery was first reported. Here we provided the evidence that shows ICP35 functions as a nuclease. Homology modelling of ICP35 showed that ICP35 may adopt a similar structure to that of TREX1 and that the protein might dimerize the same manner as seen in TREX1. While the DEDD motif is crucial for nuclease activity of TREX1, this motif is absent in ICP35. Instead, it was shown that Asn126 and Asp132, the residues located near by the metal ions, were important for nuclease activity in ICP35. This implies that although ICP35 has a similar structure to that of TREX1, the protein may employ a different mechanism for DNA digestion. However, it is still unclear what role ICP35 plays in the WSSV life cycle. Having detected as early as at 2 h p.i. indicates the possible role of ICP35 in viral replication [7]. TREX1 from humans was reported to be involved in the DNA editing and repairing process. In several studies, it has been shown that TREX1 interacts with DNA polymerases to facilitate the polymerase accuracy [21, 22]. Since the silencing of *ICP35* gene by dsRNA reduced the viral copy number in the WSSV-infected shrimp, it has been speculated that the role of ICP35 might be involved in viral replication process [8]. The localization of ICP35 in shrimp nuclease also supports this view [7]. In addition, transcriptional analysis of WSSV DNA polymerase gene (WSSV *dnapol*) and *ICP35* showed that both gene transcripts were detected during the same period in WSSV infection [23], it could be that ICP35 may play a role in DNA replication, possibly by enhancing the accuracy of the DNA polymerase. However, it will be interesting to see how silencing of ICP35 encoding gene affects the mutation rate of the virus. In summary, as far as current evidence is concerned, the similar structure to TREX1’s, the localization in the nuclease, the expression in the early stage of infection and the reduction of the viral copy number in respond to the gene silencing, these are likely to suggest that the function of ICP35 is involved in the replication of WSSV.
